# Generalized Papulovesicular Eruption as a Side Effect of the Pfizer-BioNTech COVID-19 Vaccine

**DOI:** 10.7759/cureus.22414

**Published:** 2022-02-20

**Authors:** Awadh Alamri, Yara Alghamdi, Safa J Alamri, Malak K Althaqafi, Wasan AlQurashi, Bader A Bader, Abdulrahman T Mohanna, Bashaer Almahdi

**Affiliations:** 1 Dermatology, King Abdulaziz Medical City, Jeddah, SAU; 2 College of Medicine, King Saud Bin Abdulaziz University for Health Sciences, Jeddah, SAU; 3 College of Medicine, King Abdullah International Medical Research Center, Jeddah, SAU; 4 Dermatology, King Abdullah International Medical Research Center, Jeddah, SAU; 5 General Practice, King Abdulaziz Hospital, Ministry of Health, Makkah, SAU; 6 Dermatology, King Abdulaziz University Hospital, Jeddah, SAU

**Keywords:** papulovesicular eruption, papulovesicular, vaccine, bnt162b2 mrna vaccine, bnt162b2 vaccine, pfizer vaccine, pfizer-biontech covid-19 vaccine, dermatology, cutaneous eruption, covid-19

## Abstract

COVID-19 is inflicted by SARS-CoV-2 and resulted in a global health crisis that necessitated the urgency of vaccine development to prevent its spreading among the public. Pfizer-BioNTech COVID-19 is one of the emergency use authorized (EUA) vaccines. This vaccine is efficacious against the SARS-CoV-2 virus; nonetheless, recipients have frequently reported side effects. Recipients of this vaccine experienced miscellaneous side effects like fatigue and headache. However, cutaneous eruptions of varying degrees of severity and involvements have been manifesting post-vaccination. Dermatological eruptions following vaccination against COVID-19 disease are poorly recognized. Dermatological manifestations triggered post-vaccination differ in the clinical context and patient's demographic features. The only constant factor is various clinical and histopathological relations to establish the diagnosis of cutaneous eruption post-vaccination.

Herein, we report a case of an 18-year-old male with T-cell acute lymphocytic lymphoma (ALL) in remission since August 2018 and other comorbidities. After the administration of the first dose of the Pfizer-BioNTech COVID-19 vaccine, the patient developed pruritic eczematous eruption presenting as grouped erythematous-violaceous papulovesicular lesions with fine scales over his upper and lower extremities. These eruptions started two days after the administration of the vaccine. This eruption became generalized 21 days after receiving the second dose of the Pfizer-BioNTech COVID-19 vaccine. Clinical suspicion of the drug-induced vesicular eruption was suspected; thus, a biopsy was obtained and showed erosions and mixed inflammatory cell infiltrate. From a clinical and histopathological correlation, vesicular eruption following vaccination with Pfizer-BioNTech COVID-19 was confirmed. Nevertheless, other diagnoses cannot be ruled out, but from the clinical-histopathological association, the vaccine-inflicted eruption is the likely culprit. Reports are crucial to understanding the nature of such dermatological manifestation after emerging diseases and counteractions like vaccinations. The dermatological manifestations are vaguely recognized; thus, by reporting on the cases similar to the case in this report, more data will be available to understand the nature and underlying cause of such eruptions.

## Introduction

Coronavirus (COVID-19) pandemic caused a global health catastrophe that necessitated the urgency of developing methods to prevent its spreading and impaction on health. Vaccination is one of the important courses of action for such emerging diseases. Due to the demand for vaccines against COVID-19, a variety of vaccines have undergone emergency use authorization (EUA) to be administered to the public. One of those vaccines is the Pfizer BioNTech COVID-19 messenger RNA (mRNA) (BNT162b2), which underwent EUA on December 12, 2020, in the United States. This is a nucleoside-modified mRNA vaccine that is formulated in lipid-nanoparticles and encodes the spike-glycoproteins of SSARS-CoV-2 [1].

Pfizer-BioNTech COVID-19 vaccine has proven to be 95% efficacious in decreasing symptomatic COVID-19 infection in SARS-CoV-2 seronegative individuals It decreased hospitalization and reduced the risk of severe outcomes among vaccinated individuals. Pfizer-BioNTech COVID-19 vaccine is deemed to be safe for administration to the public as the commonly observed side effects are of self-limited nature [1]. However, adverse reactions, ranging in severity, were commonly reported among vaccine recipients. There is an emergence in reporting of cutaneous manifestations after COVID-19 vaccination; however, these eruptions should not discourage the population from receiving the vaccine unless there is an absolute contraindication, like anaphylaxis [1, 2]. COVID-19 disease could elicit cutaneous manifestations, like vesicular or maculopapular eruption, necrotic lesions, urticaria, and chilblains-like lesions [2, 3]. Interestingly, vaccination against COVID-19 could induce, to some extent, similar cutaneous eruptions, such as delayed large local reactions “COVID arm”, local injection site reaction, urticarial rash, morbilliform eruptions, erythromelalgia, and cosmetic filler reaction [2-4]. The frequent clinical eruptions after COVID-19 vaccination are papules surrounded by crust-formation and pityriasis rosea-like eruption. Histopathological findings that were commonly reported are spongiotic dermatitis and mixed-cell-infiltrate with eosinophils [4-6]. The diagnosis of such dermatologic eruptions post-vaccination is based on clinical and histopathological correlation. This is a report of an 18-year-old male who developed papulovesicular eruption following first dose vaccination with Pfizer-BioNTech COVID-19, and this eruption had worsened and became generalized after second-dose vaccination.

## Case presentation

**Figure 1:**
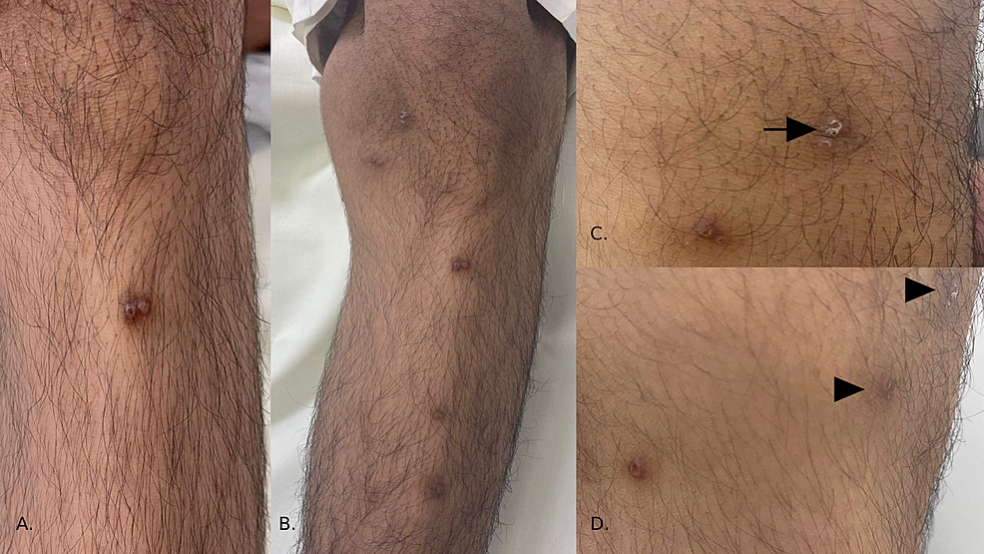
Pruritic erythematous-violaceous grouped papulovesicular lesions over acral sites. (A) Over the shin of the left leg, grouped erythematous-to-violaceous colored smooth eroded papules with hemorrhagic crust over a background of hyperpigmentation can be seen (B). Over the shin of the right leg, there are scattered post-inflammatory hyperpigmented (PIH) lesions. (C) Under the right knee, there are eroded hyper-pigmented papules. The arrow points to scales over a background of PIH. (D) Over the lateral aspect of the right knee, there is a solitary erythematous eroded papule. The arrowheads point to PIH.

**Figure 2:**
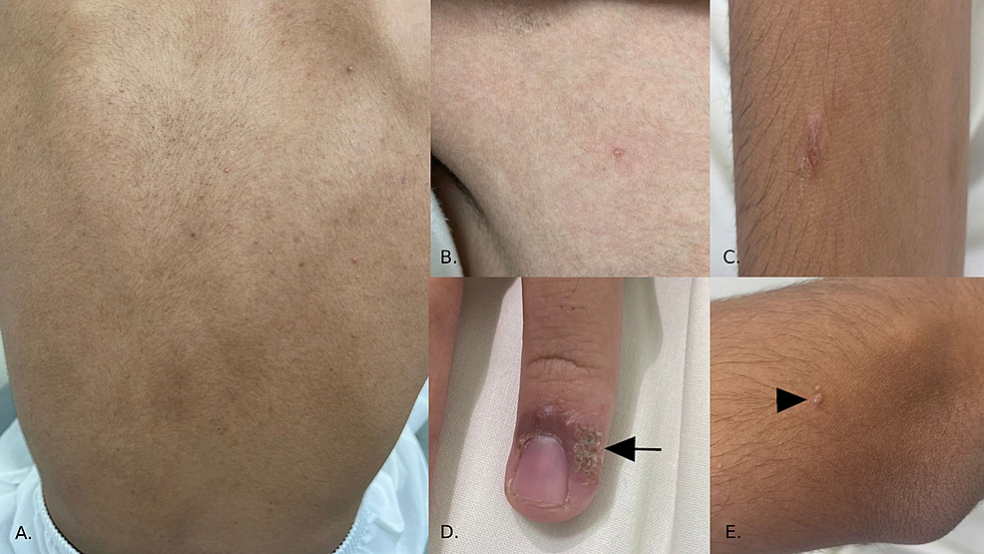
Acral eruption progressed into generalized erythematous-violaceous smooth papulovesicular lesions (A) Over the back, there are fine and small scattered erythematous-violaceous vesicles and pustules. (B) Over the right upper quadrant of the chest, there is a solitary erythematous vesicle. (C) Over the lateral aspect of the right arm, there is a solitary raised erythematous plaque with fine scales. (D) Over the periungual area of the left fourth digit, there is an erythematous-violaceous plaque with yellowish crustation (see arrow). (E) Over the extensor surface of the left forearm, there are grouped scaly erythematous papulovesicular lesions with fine scales (see arrowhead).

An 18-year-old male with a known case of T-cell acute lymphocytic lymphoma (ALL), in remission since August 2018, delayed puberty, short stature with delayed bone-age, growth-hormone deficiency, left-hip avascular necrosis, and sensorineural hearing loss, developed pruritic erythematous-violaceous grouped papulovesicular eruption with fine scales over upper and lower extremities two days after receiving first dose of the Pfizer vaccine (Figure 1). There were no face, mucosal, or systemic manifestations. Any previous history of similar lesions, prodromal symptoms, and intake of new medication(s) were all denied. The patient had received the first dose of the vaccine two days prior to the acral eruption. The second dose administration was scheduled 21 days after the first dose. Two days after receiving the second dose, the eruption had worsened and became generalized. The acral eruption progressed into generalized small pruritic erythematous-violaceous smooth papulovesicular eruption with excoriation over the chest, back, and trunk two days after second-dose administration (Figure 2). A clinical suspicious of drug-inflicted vesicular eruption after the Pfizer-BioNTech COVID-19 vaccine was considered; thus, a biopsy was obtained. The histopathological report showed erosions with mixed inflammatory cell infiltrate. From clinical-histopathological correlation, vesicular eruption following Pfizer-BioNTech COVID-19 vaccine was established. Management was symptomatic with oral antihistamine tablets (loratadine 10mg), topical mometasone furoate cream, and fusidic acid cream. On the next follow-up after a month on the topical regimen, the patient’s vesicular eruption had improved.

## Discussion

Pfizer-BioNTech COVID-19 vaccine had consistent efficacy in preventing symptomatic SARS-CoV-2 infection. The consistent data was observed among participants' age, gender, race, ethnic background, and previous exposure to the infection [1]. The volume of data available about this vaccine is one clinical trial that enrolled more than 40,000 recipients (median age = 52 years, range 16-91 years) and was conducted within a two-month duration [1]. It was deduced that the Pfizer BioNTech COVID-19 vaccine is efficacious in preventing COVID-19 infection. The commonly reported side effects are fatigue (4.2%), headache (2.4%), muscular pain (1.8%), chills (1.7%), and injection site pain (1.4%) [1]. Serious adverse events like anaphylaxis were reported outside the clinical trial and estimated to be 21 cases reported to the Center for Disease Control and Prevention (CDC) with no mortality [2, 3]. Systemic reactions were frequent after second dose vaccination, with females being more likely to develop vaccine side effects, and a severe outcome was observed in individuals aged 18-55 years [1, 2]. Adverse vaccine reactions were noticed among vaccinated persons rather than placebo group, nonetheless, the benefits outweigh the risks in preventing COVID-19 disease transmission and decreasing hospitalization [1].

Cutaneous reactions post-vaccination against COVID-19 disease could occur and are reported in the literature, but they are poorly recognized and categorized. McMahone et al. have proposed an acronym “V-REPP” (vaccine-related eruptions of papules and plaques) for the spectrum of such dermatologic manifestation [4]. The most-reported clinical manifestations post-vaccination against SARS-CoV-2 are crusted papules, pityriasis-rosea like eruptions, and pink papules with fine scales. Among the frequently encountered histopathologic finding is spongiotic dermatitis [4-6]. Clinical-histopathologic correlation is crucial to understanding the causation relationship between COVID-19 vaccination and cutaneous manifestation. There are only case reports and case-based registries that studied and discussed dermatological symptomatology after the COVID-19 vaccination. These cases ranged from pityriasis rosea-like eruptions, papulosquamous, to papulovesicular eruptions, which all were clinically and histopathologically classified as vaccine-related exanthems, or V-REPP [4-10]. The patient in this report developed pruritic erythematous-to-violaceous vesicles and papules with scales that coalesce into plaques, and manifested in acral areas initially after first-dose administration, and then worsened to be generalized after second-dose vaccination. Histopathology showed mixed inflammatory-cell infiltrate. Thus, the likely dermatological diagnosis is papulovesicular eruption secondary to vaccination with Pfizer-BioNTech COVID-19. Other clinical conditions could not be ruled out; however, with clinical-and-histopathological correlation, the vaccine-induced vesicular eruption is the likely culprit in such manifestation.

Vaccines are drugs that achieve protective immunity against an antigen. The cutaneous adverse reactions range from localized to generalized (self-limiting or life-threatening) manifestations. The histopathology could show changes in the epidermis, dermo-epidermal junction, upper dermis, or sub-cutis. COVID-19 vaccines differ in their manufacture, like mRNA-based (e.g., Pfizer-BioNTech COVID-19 vaccine) or vector-based (e.g., Oxford-AstraZeneca vaccine). The cutaneous reactions differ among recipients of those vaccines as they are manufactured differently [11].

As reactions could differ, papulovesicular eruptions had been reported before twice, and in both cases occurred after Pfizer-BioNTech COVID-19 vaccination [11]. The first was a case report of a 55-year-old female, with a history of atopic diathesis and chronic spontaneous urticaria, who developed a vesicular reaction after second-dose administration of Pfizer-BioNTech COVID-19 vaccine. The grouped-pruritic papulovesicular eruption manifested seven days post-vaccination. Management was symptomatic with fusidin ointment twice daily and resulted in an excellent outcome [11]. The second report was of a healthy 49-year-old female who developed vesicular eruption after second dose vaccination with Pfizer-BioNTech COVID-19 vaccine. In this case, the grouped-pruritic papulovesicular eruption manifested three days after vaccination. However, her first-dose vaccination was the Oxford-AstraZeneca vaccine. Symptomatic management was implemented with topical corticosteroids twice daily which resulted in an excellent outcome [11].

The cases mentioned above of vesicular eruption post-vaccination are comparable to the present case report in the manner of papulovesicular temporal onset and improvement with topical therapy. However, the histopathology findings of the two previously reported cases were unavailable. The significant differences in age and medical comorbidities between the patients in the previously reported cases and this case report were noted. Thus, eruptions post-vaccination are heterogenous in the manner of age, dose-related eruption, the onset of lesions, and medical comorbidities. The consistent factor is a clinical diagnosis in the absence of histopathological evaluation.

## Conclusions

COVID-19 vaccine drug reactions, specifically those involving dermatologic eruptions, are vaguely recognized. Most of the available data are from case reports. Thus, reporting adverse events, specifically those of cutaneous manner, is crucial to ease the process of categorization and diagnosis. Specific demographic factors, the dose-related onset of eruptions, medical history, and comorbidities are all of great significance in determining the predicting factors of dermatological manifestation. However, clinical and histopathological findings were consistent among cases of cutaneous eruption post-vaccination; thus, diagnosis is dependent on the correlation of those two consistent factors. 

## References

[1] Oliver SE, Gargano JW, Marin M (2020). The advisory committee on immunization practices' interim recommendation for use of Pfizer-BioNTech COVID-19 vaccine - United States, December 2020. MMWR Morb Mortal Wkly Rep.

[2] McMahon DE, Amerson E, Rosenbach M (2021). Cutaneous reactions reported after Moderna and Pfizer COVID-19 vaccination: A registry-based study of 414 cases. J Am Acad Dermatol.

[3] Corbeddu M, Diociaiuti A, Vinci MR, Santoro A, Camisa V, Zaffina S, El Hachem M (2021). Transient cutaneous manifestations after administration of Pfizer-BioNTech COVID-19 Vaccine: an Italian single-centre case series. J Eur Acad Dermatol Venereol.

[4] McMahon DE, Kovarik CL, Damsky W (2022). Clinical and pathologic correlation of cutaneous COVID-19 vaccine reactions including V-REPP: A registry-based study. J Am Acad Dermatol.

[5] Larson V, Seidenberg R, Caplan A, Brinster NK, Meehan SA, Kim RH (2022). Clinical and histopathological spectrum of delayed adverse cutaneous reactions following COVID-19 vaccination. J Cutan Pathol.

[6] Niebel D, Novak N, Wilhelmi J (2021). Cutaneous adverse reactions to COVID-19 vaccines: insights from an immuno-dermatological perspective. Vaccines (Basel).

[7] Kim MJ, Kim JW, Kim MS, Choi SY, Na JI (2022). Generalized erythema multiforme-like skin rash following the first dose of COVID-19 vaccine (Pfizer-BioNTech). J Eur Acad Dermatol Venereol.

[8] Buján Bonino C, Moreiras Arias N, López-Pardo Rico M, Pita da Veiga Seijo G, Rosón López E, Suárez Peñaranda JM, Sánchez-Aguilar Rojas D (2021). Atypical erythema multiforme related to BNT162b2 (Pfizer-BioNTech) COVID-19 vaccine. Int J Dermatol.

[9] Abdullah L, Hasbani D, Kurban M, Abbas O (2021). Pityriasis rosea after mRNA COVID-19 vaccination. Int J Dermatol.

[10] Cyrenne BM, Al-Mohammedi F, DeKoven JG, Alhusayen R (2021). Pityriasis rosea-like eruptions following vaccination with BNT162b2 mRNA COVID-19 Vaccine. J Eur Acad Dermatol Venereol.

[11] Niebel D, Wenzel J, Wilsmann-Theis D, Ziob J, Wilhelmi J, Braegelmann C (2021). Single-center clinico-pathological case study of 19 patients with cutaneous adverse reactions following COVID-19 vaccines. Dermatopathology (Basel).

